# Land‐Use Effects on Melliferous Woody Flora in Sudanian Agroforestry Parkland and Protected Area of West Africa

**DOI:** 10.1002/ece3.73555

**Published:** 2026-04-21

**Authors:** Issaka Wendpanga Kanazoe, Issa Nombré, Adama Zoungrana, Joseph Issaka Boussim

**Affiliations:** ^1^ Laboratoire de Biologie et Écologie Végétales Université Joseph KI‐ZERBO Ouagadougou Burkina Faso; ^2^ Centre Universitaire de Manga Université Norbert Zongo Koudougou Burkina Faso; ^3^ École Normale Supérieure, Institut des Sciences et Technologie Ouagadougou Burkina Faso; ^4^ Centre Universitaire de Ziniaré Université Joseph KI‐ZERBO Ouagadougou Burkina Faso

**Keywords:** agroforestry systems, *Apis mellifera* (honeybee) foraging, biodiversity conservation, Burkina Faso, floral resource availability, sustainable management

## Abstract

Land‐use intensification alters vegetation structure and composition, with potential consequences for pollinator‐dependent ecosystems in tropical regions. However, the distribution and availability of melliferous woody flora across contrasting land use types in West Africa remain poorly studied. This study evaluated melliferous floristic composition and woody stand structure across sudanian agroforestry parkland (farmland and fallow land) and protected area in southwestern Burkina Faso. Ninety vegetation plots were surveyed, and flowering dynamics were monitored monthly over 1 year. A total of 48 melliferous woody species belonging to 16 families were recorded, dominated by Fabaceae, Combretaceae, and Sapotaceae. Species composition differed significantly among land use types. Fallow land and protected area supported the highest melliferous species richness, whereas farmland exhibited reduced richness and density but larger individual trees. *Vitellaria paradoxa* dominated all land use types. Indicator species analysis identified 19 significant melliferous species. Among these, *Combretum glutinosum*, *Terminalia avicennioides*, and *Terminalia leiocarpa* were the principal indicator species associated with both fallow land and protected area, whereas no species was identified as specific to farmland. Protected area maintained the highest melliferous tree densities and greater species evenness. Temporal patterns of flowering varied among land use types. The protected area ensured extended forage availability for a minimum of 3 months, with *Mitragyna inermis* notably flowering for up to 5 months. These results support integrated landscape management combining conservation of melliferous habitats with targeted enrichment of woody species to sustain pollination services and beekeeping productivity in West Africa.

## Graphical abstract

This study assessed how land use types influence the diversity, structure, and temporal dynamics of melliferous woody species and honeybee ( *Apis mellifera* ) foraging in a sudanian landscape of southwestern Burkina Faso. Fallow land and protected area supported higher species richness and distinct community composition compared with farmland, while protected area maintained greater tree densities, higher evenness, and longer periods of floral resource availability. These findings highlight the complementary roles of agroforestry parkland (fallow land and farmland) and protected area in sustaining honeybee forage and supporting pollination services in West Africa.

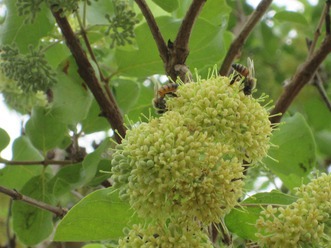

## 1 Introduction

Insect pollination, particularly by the honeybee ( *Apis mellifera* ), represents a key ecosystem service supporting both agricultural production and the functioning of natural ecosystems (Klein et al. 2007). As one of the many pollinators of both wild and cultivated plant species, *A. mellifera* plays an important role in food security, ecosystem stability, and biodiversity conservation (Hung et al. 2018). This ecological role is particularly important in tropical regions, where plant‐pollinator interactions are more complex and diverse than in temperate zones (Bawa 1990; Olesen and Jordano 2002; Vizentin‐Bugoni et al. 2018).

In West African savannas, beekeeping activities are largely supported by traditional agroforestry parkland and protected area (Kanazoe and Nombré 2021). Agroforestry parkland is characterized by heterogeneous landscapes in which cultivated fields are interspersed with deliberately preserved or managed mature trees, often integrated with livestock systems. This land use type also include fallow land, where temporarily rested fields allow natural vegetation to regenerate, thereby enhancing soil fertility, biodiversity, and overall ecosystem services (Boffa 1999; Bayala et al. 2014; Zoungrana et al. 2024). Developed over centuries by farmers, agroforestry parkland represents a traditional form of integrated land management that maintains tree cover while supporting agricultural production. In contrast, protected area conserves native biodiversity and provides reference conditions for understanding the natural distribution and abundance of melliferous species (Dassou et al. 2020; Kanazoe et al. 2023; Pulido‐Chadid et al. 2023).

However, both agroforestry parkland and protected area are facing increasing pressures that threaten their ecological functions and economic value. In particular, anthropogenic drivers such as agricultural intensification, shortened fallow cycles, and unsustainable wood extraction contribute to the progressive degradation of woody species in agroforestry parkland (Zoungrana et al. 2025). Similarly, protected area struggles with encroachment, limited management resources, and the impacts of climate change (Tranquilli et al. 2014; Aleman et al. 2018). These pressures directly reduce the availability and diversity of melliferous plant species, with consequences for wild pollinators and beekeeping. Moreover, in Africa, documentation on bee forage plants is limited in West, Central, and East Africa when compared to Southern Africa (Nganso et al. 2025). Understanding variation in melliferous plant communities across land use types is therefore important for designing conservation strategies that support both biodiversity and rural livelihoods. This information can guide the placement and management of apiaries in different landscape contexts and help clarify trade‐offs among ecosystem services in multifunctional landscapes. Previous studies in West Africa have documented melliferous plants in specific contexts, such as natural forests (Nombré et al. 2009) and agricultural landscapes (Dassou et al. 2020). However, comparative analyses across contrasting land‐use types are limited, particularly regarding woody stand structure and its influence on melliferous potential. Although the effects of land‐use change on overall vegetation structure are well documented (Nacoulma et al. 2011), their specific consequences for melliferous plant communities are less well understood.

This study aims to evaluate melliferous floristic composition and woody stand structure across agroforestry parkland (fallow land and farmland) and protected area in Burkina Faso (West Africa). Specifically, this study (i) analyses the diversity of melliferous plant species across land use types; (ii) assesses the woody stand structure of melliferous plant species across land use types and (iii) determines the temporal dynamics of floral resource availability for honeybee foraging across land use types. Three main hypotheses were tested. (i) Protected area maintains higher melliferous plant species richness than agroforestry parkland. This was evaluated by comparing species diversity across land use types. (ii) Woody stand structure of melliferous plant communities differs among land use types, reflecting variation in land use intensity and management practices. Structural parameters were therefore measured within each land use type. (iii) The temporal availability of floral resources for honeybee varies among land use types, with protected area expected to support species with higher densities and longer flowering periods. This was assessed by quantifying, for each land‐use type, the monthly number of flowering melliferous species and their density.

## 2 Materials and Methods

### 2.1 Study Area

**FIGURE 1:**
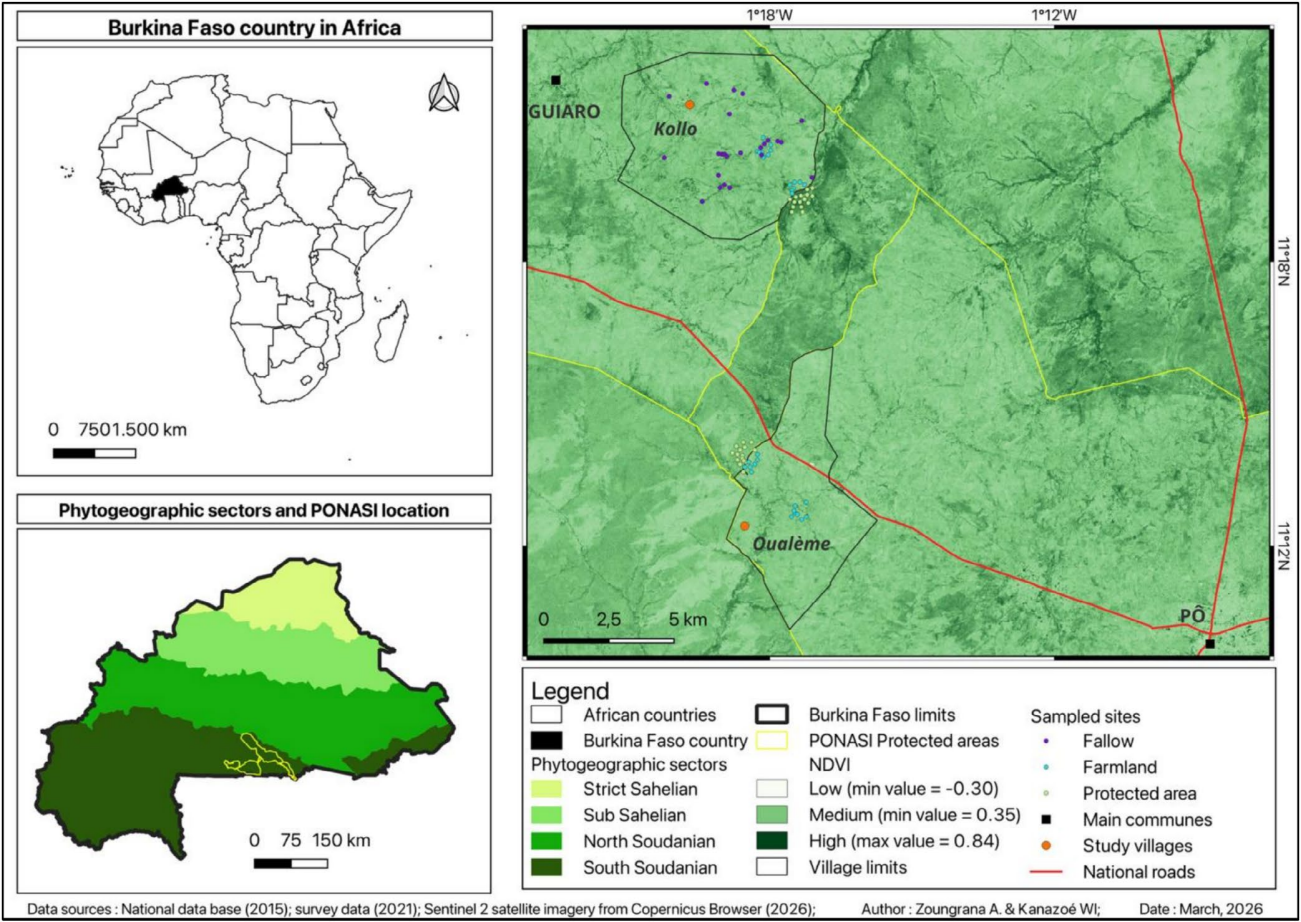
Study area and sampling design. Yellow points within the PONASI boundary (yellow outline) indicate protected area sampling plots, while those within village territories (purple and blue points) indicate agroforestry parkland (farmland and fallow land) sampling plots.

The study was conducted in southwestern Burkina Faso (Figure 1), within the Sudanian phytogeographical zone. This region receives an average annual rainfall of 1012 mm, with a distinct rainy season (June–October) and dry season (November–May). The mean annual temperature is 28.61°C. The study area includes a complex of protected areas, notably the Pô‐Nazinga‐Sissili (PONASI) protected area, as well as surrounding agroforestry systems (Zoungrana, De Cannière, et al. 2023). The Normalized Difference Vegetation Index (NDVI) of the study area ranges from −0.30 to +0.84, indicating a gradient from low to high vegetation cover in the context of the study area. Areas with higher NDVI values correspond to wooded savannas and gallery forests, which are predominantly located within the PONASI protected area (Sama 2019; Zoungrana, Visser, et al. 2023).

### 2.2 Data Collection

Floristic surveys focused on adult individuals within each sampling plot. All woody plants with a stem diameter ≥ 5 cm measured at 1.5 m above ground were recorded. Lianas with a stem diameter ≥ 5 cm measured at 20 cm above ground were also included. For each individual, stem diameter and total height were measured. A total of 90 plots were established across three land use types (protected area, fallow land, and farmland), with 30 plots per land use type. Plots were distributed both upstream and downstream of the PONASI protected area. Plot size followed standard vegetation sampling protocols for West Africa and was adapted to land use type (Thiombiano et al. 2015). Surveys covered 2500 m^2^ (50 × 50 m) in farmland and fallow land, and 900 m^2^ (30 × 30 m) within protected area (Thiombiano et al. 2015). Within protected area, plots were randomly located within a 1 km radius of apiaries near the boundary. In farmland and fallow land, plots were also randomly established around apiaries located within each land use type. The mean duration of fallowing was 16.5 ± 7.9 years.

The list of melliferous species was compiled from previous studies conducted in the region and validated through consultations with local beekeepers (Kanazoe et al. 2023). Flowering melliferous species occurring near apiaries were recorded monthly from January to December 2021 through direct observations of *Apis mellifera* foraging activity (Nombré et al. 2001; Yédomonhan et al. 2009). Observations were conducted at four experimental sites following a protocol adapted from Ahouandjinou et al. (2017). Inventories were carried out over two consecutive days each month during peak foraging periods (05:00–09:00 and 15:00–18:00).

### 2.3 Data Analysis

All statistical analyses were performed in R (R Core Team 2024). Differences in melliferous plant species communities among land use types were examined using non‐metric multidimensional scaling (NMDS). Indicator species were identified with the Indicator Value (IndVal) method of Dufrêne and Legendre (1997), and the ecological importance of each species was quantified using the Importance Value Index (IVI). Generalized linear models (GLMs), followed by Tukey post hoc tests, were used to compare woody stand structure across land use types. Poisson error distributions were applied to count data (tree density), whereas Gamma error distributions were used for positively skewed continuous variables (other structural parameters). The structural parameters considered are presented in Table 1. Differences in diversity indices (species richness, Shannon index, and Pielou evenness) among land use types were assessed using Kruskal‐Wallis tests followed by Dunn's post hoc tests. NMDS analyses were conducted using the vegan package (Oksanen et al. 2022). Indicator values were calculated with the *indicspecies* package (Cáceres and Legendre 2009), while IVI, structural parameters, and diversity indices were computed using *BiodiversityR* (Kindt and Kindt 2019). Figures were produced with *ggplot2* (Wickham 2016). Statistical significance was set at 5%.

**TABLE 1:** Woody stand structure parameters. N (trees/ha): Tree density; n: Total number of trees per plot; s: Plot area; D (cm): Mean diameter; dᵢ: Sum of diameters; H (m): Mean height; hᵢ: Sum of heights; G (m^2^/ha): Basal area.

Parameters	Formula
Density (*N*)	$N=\frac{n}{s}\times 10000$
Mean diameter (*D*)	$D=\frac{1}{n}\sum_{i=1}^{n}d_{i}$
Mean height (*H*)	$H=\frac{1}{n}\sum_{i=1}^{n}h_{i}$
Basal area (*G*)	$G=\frac{\pi }{40000S}\sum_{i=1}^{n}{\mathrm{di}}^{2}$
Relative dominance (Rel.Do)	$\mathrm{Rel}.\mathrm{Do}=\frac{\text{Basal area of the species}}{\text{Basal area of}\;\mathrm{all}\;\text{species}\;}\times 100$
Relative density (Rel.den)	$\mathrm{Rel}.\mathrm{den}=\frac{\text{Number of individuals of the species}\;\mathrm{per}\;\mathrm{ha}}{\text{Total number of individuals}\;\mathrm{per}\;\mathrm{ha}\;}\times 100$
Frequency (Freq)	$\text{Freq}=\frac{\text{Number of plots in which}\;a\;\text{species is found}}{\text{Total number of plots}\;}\times 100$
Relative frequency (Rel.freq)	$\mathrm{Rel}.\text{freq}=\frac{\text{Frequency of the species}\;}{\mathrm{Sum}\;\text{of the species frequencies}\;}\times 100$
Importance Value Index (IVI)	IVI = Relative dominance + Relative density + Relative frequency

## 3 Results

### 3.1 Composition of Melliferous Plant Communities Across Land Use Types

**FIGURE 2:**
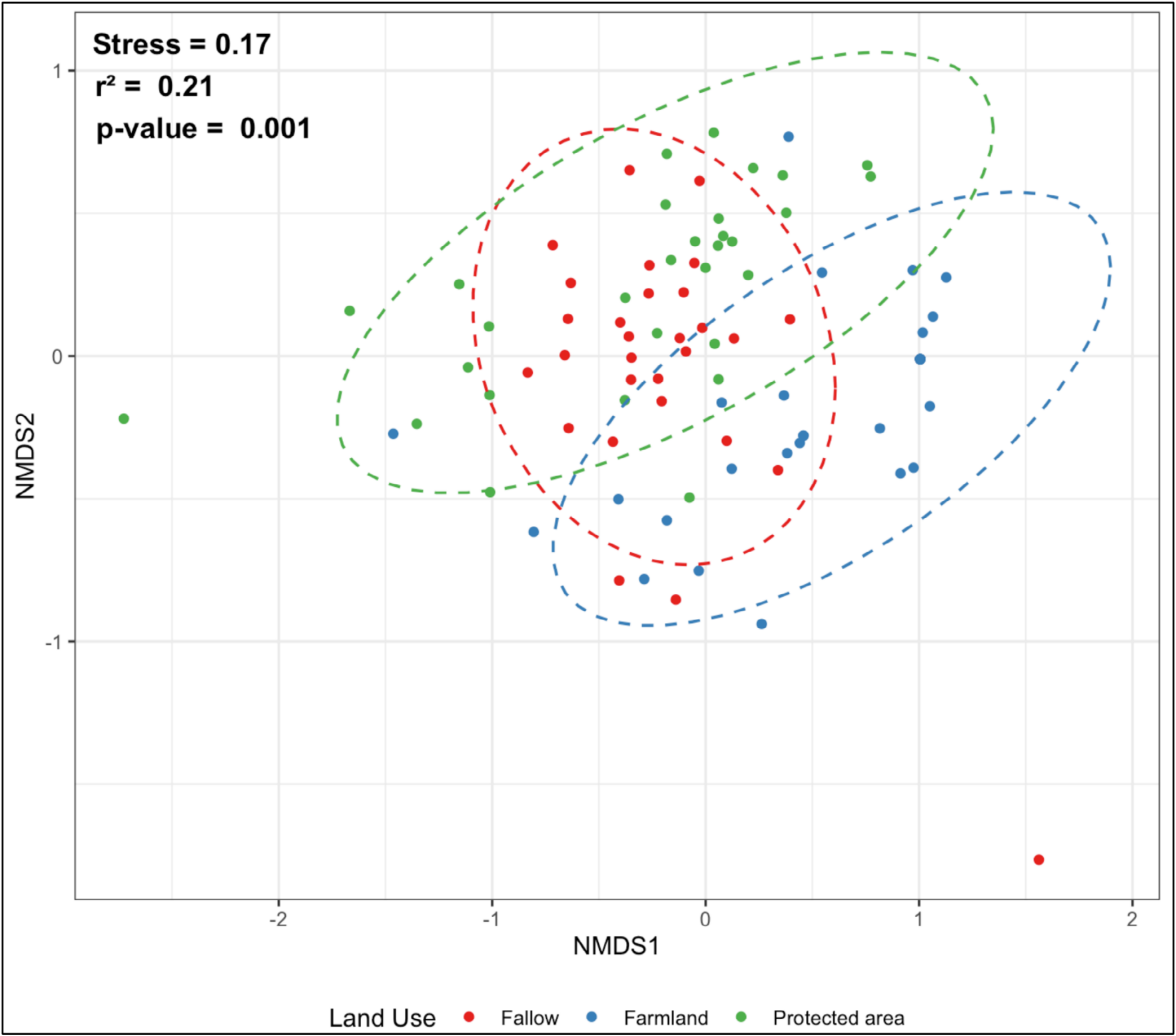
Non‐metric multidimensional scaling ordination of melliferous plant communities across land use types. Points represent sampling plots and are color‐coded according to land use type. The reported *r* ^2^ and *p*‐value were derived from the *envfit* function (*vegan* R package).

**FIGURE 3:**
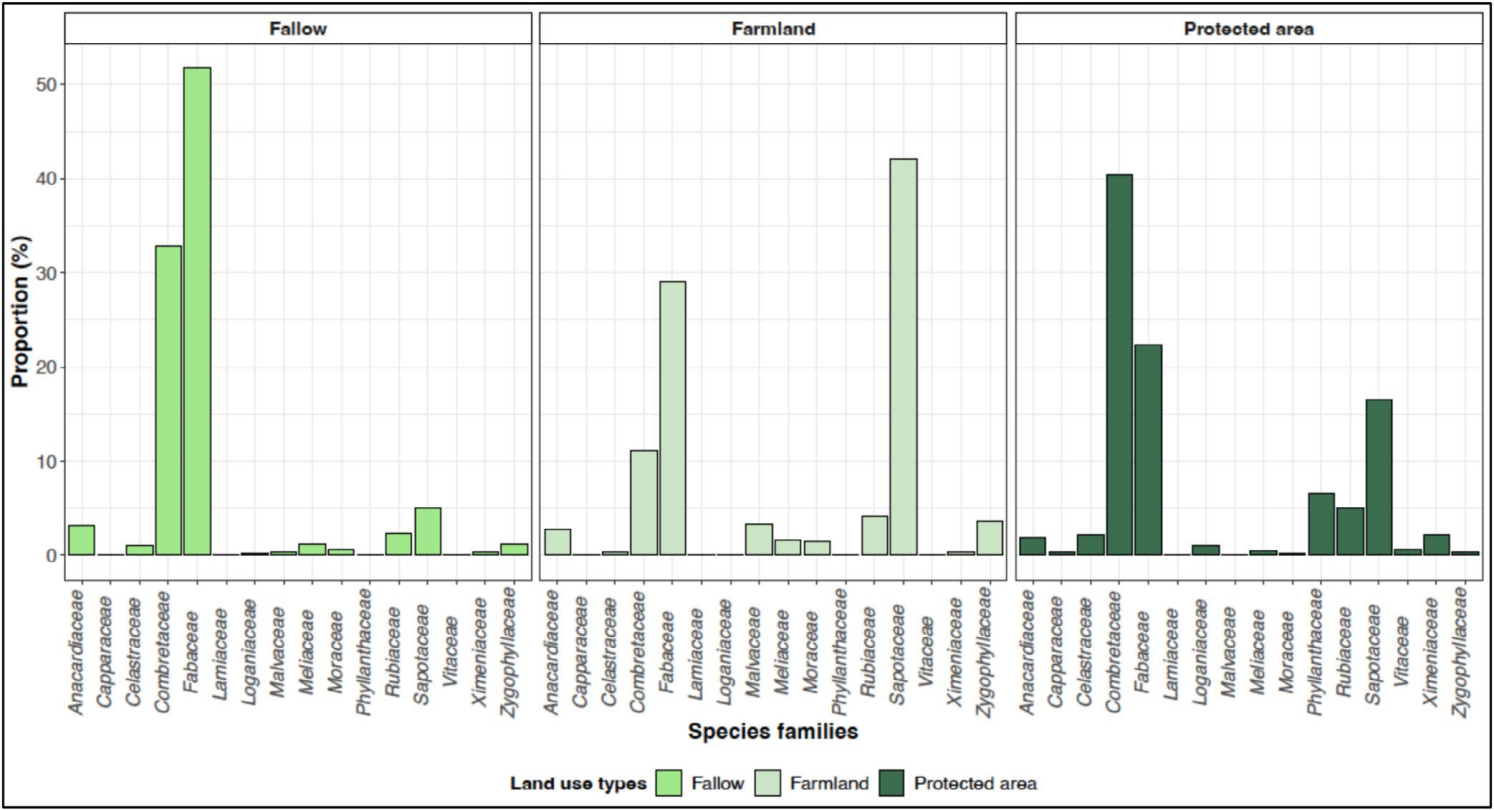
Spectrum of melliferous plant families across land use types.

Across all land use types, the floristic survey recorded 48 melliferous woody species, 35 genera and 16 families (Table 2). Fallow land comprised 42 species belonging to 32 genera and 15 families. Farmland included 36 species (27 genera, 11 families), while the protected area supported 35 species (26 genera, 14 families) (Table 2). Non‐metric multidimensional scaling (NMDS) indicated significant differences in species communities among land use types (Stress = 0.174; *r* ^*2*^ = 0.214; *p* < 0.05; Figure 2). Fabaceae (41.12% for all land use types), Combretaceae (32.12% for all land use types), and Sapotaceae (12.69% for all land use types) are the main families of melliferous plant species recorded across all land use types (Figure 3). Although these three families occur in all land use types, Fabaceae show the highest proportion in fallow land (51.71%), Sapotaceae are most represented in farmland (42.17%), whereas Combretaceae dominate in protected area (40.42%) (Figure 3). Regarding the diversity indices of melliferous plants, farmland exhibited significantly lower species richness and Shannon index (*p* < 0.05) compared to fallow land and protected area, which did not differ significantly from each other (Table 3). In addition, Pielou's evenness index differed significantly between fallow land and protected area (*p* < 0.05; Table 3).

**TABLE 2:** Melliferous plant surveyed across land use types. + = presence, − = absence.

Species	Genera	Families	Fallow	Farmland	Protected area
*Adansonia digitata* L.	Adansonia	Malvaceae	+	+	−
*Albizia chevalieri* Harms	Albizia	Fabaceae	+	−	+
*Balanites aegyptiaca* (L.) Delile	Balanites	Zygophyllaceae	+	+	+
*Bombax costatum* Pellegr. & Vuillet	Bombax	Malvaceae	+	+	−
*Cissus populnea* Guill. & Perr.	Cissus	Vitaceae	+	−	+
*Combretum adenogonium* Steud. ex A.Rich.	Combretum	Combretaceae	+	+	+
*Combretum collinum* Fresen.	Combretum	Combretaceae	−	+	+
*Combretum glutinosum* Perr. ex DC.	Combretum	Combretaceae	+	+	+
*Combretum paniculatum* Vent.	Combretum	Combretaceae	−	+	+
*Crateva adansonii* DC.	Crateva	Capparaceae	−	−	+
*Crossopteryx febrifuga* (Afzel. ex G.Don) Benth.	Crossopteryx	Rubiaceae	+	+	+
*Daniellia oliveri* (Rolfe) Hutch. & Dalziel	Daniellia	Fabaceae	+	+	+
*Detarium microcarpum* Guill. & Perr.	Detarium	Fabaceae	+	+	+
*Entada africana* Guill. & Perr.	Entada	Fabaceae	+	+	−
*Feretia apodanthera* Delile	Feretia	Rubiaceae	+	+	+
*Ficus sycomorus* L.	Ficus	Moraceae	+	+	+
*Flueggea virosa* (Roxb. ex Willd.) Royle	Flueggea	Phyllanthaceae	+	−	+
*Gardenia erubescens* Stapf & Hutch.	Gardenia	Rubiaceae	+	+	+
*Grewia flavescens* Juss.	Grewia	Malvaceae	−	+	−
*Grewia lasiodiscus* K.Schum.	Grewia	Malvaceae	+	−	−
*Gymnosporia senegalensis* (Lam.) Loes.	Gymnosporia	Celastraceae	+	+	+
*Khaya senegalensis* (Desr.) A.Juss.	Khaya	Meliaceae	−	+	+
*Lannea acida* A.Rich.	Lannea	Anacardiaceae	+	+	+
*Lannea microcarpa* Engl. & K.Krause	Lannea	Anacardiaceae	+	+	+
*Lannea velutina* A.Rich.	Lannea	Anacardiaceae	+	+	+
*Mitragyna inermis* (Willd.) Kuntze	Mitragyna	Rubiaceae	+	+	+
*Nauclea latifolia* Sm.	Nauclea	Rubiaceae	+	+	+
*Ozoroa insignis* Delile	Ozoroa	Anacardiaceae	+	−	−
*Parkia biglobosa* (Jacq.) R.Br. ex G.Don	Parkia	Fabaceae	+	+	−
*Piliostigma thonningii* (Schumach.) Milne‐Redh.	Piliostigma	Fabaceae	+	+	+
*Pseudocedrela kotschyi* (Schweinf.) Harms	Pseudocedrela	Meliaceae	+	+	+
*Senegalia dudgeonii* (Craib) Kyal. & Boatwr.	Senegalia	Fabaceae	+	+	+
*Senegalia gourmaensis* (A.Chev.) Kyal. & Boatwr.	Senegalia	Fabaceae	+	+	+
*Senegalia polyacantha* (Willd.) Seigler & Ebinger	Senegalia	Fabaceae	+	+	+
*Sterculia setigera* Delile	Sterculia	Malvaceae	+	+	−
*Strychnos spinosa* Lam.	Strychnos	Loganiaceae	+	−	+
*Tamarindus indica* L.	Tamarindus	Fabaceae	+	+	−
*Terminalia avicennioides* Guill. & Perr.	Terminalia	Combretaceae	+	+	+
*Terminalia laxiflora* Engl.	Terminalia	Combretaceae	+	+	+
*Terminalia leiocarpa* (DC.) Baill.	Terminalia	Combretaceae	+	+	+
*Terminalia macroptera* Guill. & Perr.	Terminalia	Combretaceae	+	−	−
*Terminalia schimperiana* Hochst.	Terminalia	Combretaceae	+	−	−
*Trichilia emetica* Vahl	Trichilia	Meliaceae	−	−	+
*Vachellia seyal* (Delile) P.J.H.Hurter	Vachellia	Fabaceae	+	−	−
*Vachellia sieberiana* (DC.) Kyal. & Boatwr.	Vachellia	Fabaceae	+	+	+
*Vitellaria paradoxa* C.F.Gaertn.	Vitellaria	Sapotaceae	+	+	+
*Vitex doniana* Sweet	Vitex	Lamiaceae	+	−	−
*Ximenia americana* L.	Ximenia	Ximeniaceae	+	+	+

**TABLE 3:** Diversity indices of melliferous plant across land use types.

Diversity indices	Protected area (mean ± SD)	Fallow (mean ± SD)	Farmland (mean ± SD)	*W*	*p*
Species richness (*S*)	7.1 ± 2.1^a^	8.8 ± 4.3^a^	3.5 ± 2.3^b^	32.8	7.71E‐08
Shannon index (*H*)	1.59 ± 0.31^a^	1.49 ± 0.61^a^	0.80 ± 0.54^b^	28.8	5.6E‐07
Pielou evenness (*J*)	0.83 ± 0.009^a^	0.73 ± 0.14^b^	0.78 ± 0.17^ab^	7.64	0.0219

*Note: W* is the Kruskal‐Wallis rank sum statistic. *p* derived from the Kruskal–Wallis test. Superscript letters indicate significant differences according to Dunn test. Numbers with the same letter indicate no significant difference at the 5% threshold and different letters indicate significant differences at *p* < 0.05.

### 3.2 Characteristic Melliferous Plant Species Across Land Use Types

Indicator species analysis (IndVal) identified 19 significant indicator species across land use types (Table 4). Several species were strongly associated with specific land use types. *Combretum glutinosum*, *Terminalia avicennioides*, *Terminalia leiocarpa, Terminalia laxiflora*, and *Gymnosporia senegalensis* were significantly associated with both fallow land and protected area (*p* < 0.05; Table 4). *Flueggea virosa* and *Mitragyna inermis* were the principal species exclusively associated with protected area (*p* < 0.05; Table 4). Fallow land was characterized by several species such as *Lannea microcarpa*, *Senegalia gourmaensis*, and *Vachellia sieberiana*. *Balanites aegyptiaca* and *Senegalia dudgeonii* (*p* < 0.05; Table 4). No species was identified as a specific indicator of farmland (Table 4).

**TABLE 4:** Indicator species analysis of melliferous plant species across land use types.

Species	Fallow	Farmland	Protected area	Index	Stat	*p*
*Combretum glutinosum*	1	0	1	5	0.730	0.001
*Flueggea virosa*	0	0	1	3	0.550	0.001
*Lannea macrocarpa*	1	0	0	1	0.670	0.001
*Senegalia gourmaensis*	1	0	0	1	0.583	0.001
*Terminalia avicennioides*	1	0	1	5	0.712	0.001
*Vachellia seyal*	1	0	0	1	0.547	0.001
*Vachellia sieberiana*	1	0	0	1	0.596	0.002
*Balanites aegyptiaca*	1	0	0	1	0.506	0.003
*Senegalia dudgeonii*	1	0	0	1	0.557	0.005
*Terminalia leiocarpa*	1	0	1	5	0.607	0.005
*Terminalia laxiflora*	1	0	1	5	0.517	0.014
*Gymnosporia senegalensis*	1	0	1	5	0.500	0.017
*Entada africana*	1	0	0	1	0.414	0.019
*Mitragyna inermis*	0	0	1	3	0.426	0.024
*Ozoroa insignis*	1	0	0	1	0.365	0.031
*Combretum collinum*	0	0	1	3	0.387	0.041
*Ximenia americana*	0	0	1	3	0.421	0.042
*Combretum paniculatum*	0	0	1	3	0.372	0.043
*Gardenia erubescens*	0	0	1	3	0.387	0.048

*Note:* Columns Fallow, Farmland, and Protected area define the group (1 = presence, 0 = absence) for which the species is a significant indicator. Index represents the group combination code. Stat represents the association strength (IndVal statistic), ranging from 0 to 1. *p* indicates the significance of the association.

### 3.3 Ecological Importance and Distribution Patterns of Melliferous Plant Species

**FIGURE 4:**
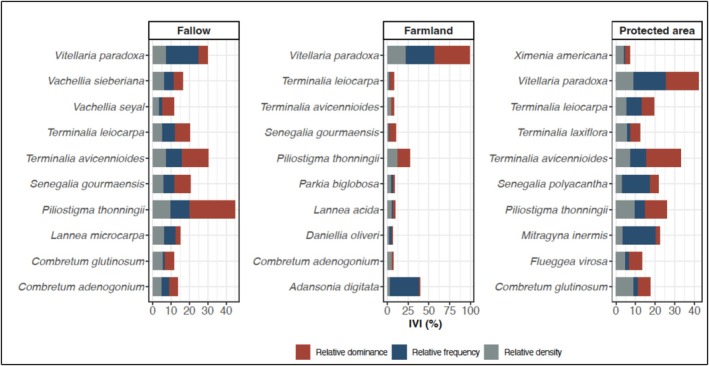
Importance value index of the top 10 melliferous plant species across land use types.

The Importance Value Index (IVI) indicated clear differences in species dominance among land use types (Figure 4). In fallow land, *Piliostigma thonningii* (44.87%), *Terminalia avicennioides* (30.33%), and *Vitellaria paradoxa* (30.06%) had the highest IVI values (Figure 4). Farmland was strongly dominated by *Vitellaria paradoxa* (99.03%), followed by *Adansonia digitata* (39.46%) and *Piliostigma thonningii* (27.27%) (Figure 4). In protected area, *Vitellaria paradoxa* (41.94%), *Terminalia avicennioides* (32.88%), and *Piliostigma thonningii* (25.96%) ranked highest in IVI values (Figure 4).

### 3.4 Woody Stand Structure and Diversity of Melliferous Plant Species Across Land Use Types

Woody stand structure differed significantly among land use types. Tree density varied across land use types (*p* < 0.05), with protected area supporting the highest density (280.4 ± 132.5 trees/ha), followed by fallow land (233.6 ± 196.1 trees/ha) and farmland (47.7 ± 43.6 trees/ha) (Table 5). Tree height also differed significantly (p < 0.05), with farmland exhibiting the greatest values (6.6 ± 2.8 m). Similarly, stem diameter varied among land use types (*p* < 0.05), with larger diameters observed in farmland compared with protected area. In contrast, basal area did not differ significantly among land use types (*p* > 0.05).

**TABLE 5:** Structural parameters of melliferous plant species.

Structural parameters	Protected area (mean ± SD)	Fallow (mean ± SD)	Farmland (mean ± SD)	*p*
Density (trees/ha)	280.4 ± 132.5^a^	233.6 ± 196.1^b^	47.7 ± 43.6^c^	6.78E‐09
Total height (m)	4.9 ± 2.3^b^	4.8 ± 1.4^b^	6.6 ± 2.8^a^	0.0043
Basal area (m^2^/ha)	4.8 ± 3.3^a^	3.1 ± 2.4^a^	3.4 ± 4.6^a^	0.151
Steam diameter (cm)	13.3 ± 8.5^c^	14 ± 10.2^b^	27.9 ± 19.5^a^	6.31E‐05

*Note:* Superscript letters indicate significant differences according to Tukey test. Numbers with the same letter indicate no significant difference at the 5% threshold.

Abbreviation: SD, standard deviation.

### 3.5 Melliferous Plant Resource Availability According to Land Use Types

**FIGURE 5:**
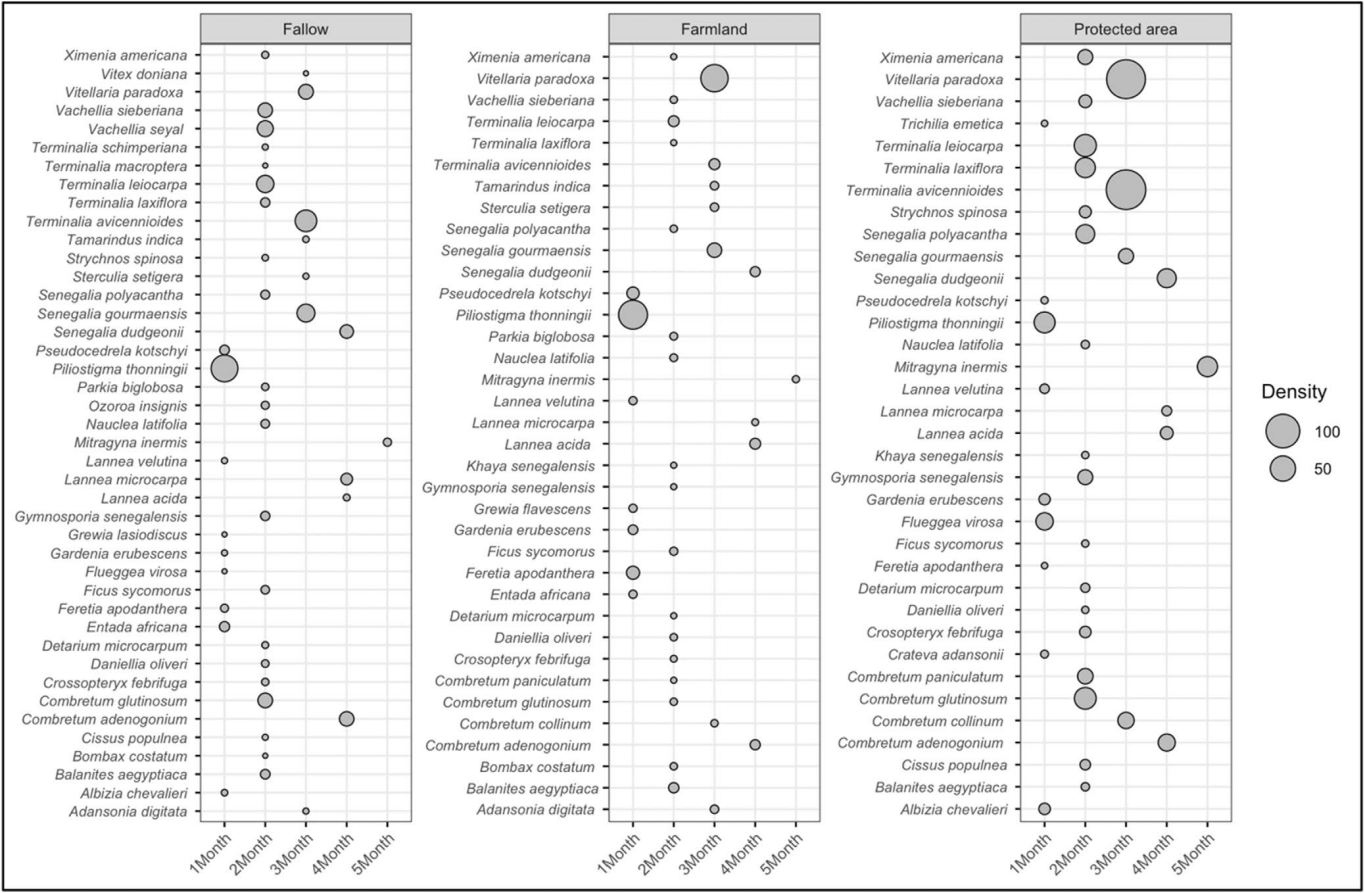
Melliferous plant species density and flowering duration across land use types.

Temporal patterns of floral resource availability varied among land use types (Figure 5). The protected area supported the highest densities of melliferous species (Figure 5). Indeed, in fallow land and farmland, the most abundant species, including *Piliostigma thonningii*, *Terminalia avicennioides*, *Terminalia leiocarpa, Senegalia gourmaensis*, and *Vitellaria paradoxa* , have flowering durations not exceeding t months (Figure 5). In contrast, within the protected area, in addition to these species, which occur at even higher densities, *Mitragyna inermis* stands out with a higher density than in farmland and fallow land and a flowering duration of at least 5 months, thereby supporting a more sustained availability of floral resources (Figure 5).

## 4 Discussion

### 4.1 Influence of Land Use on Melliferous Plant Species Richness and Distribution

A total of 48 melliferous woody species were recorded across the study landscape, confirming that sudanian agroforestry parkland and protected area both contribute substantially to honeybee forage resources. The dominance of Fabaceae, Combretaceae, and Sapotaceae families is consistent with the floristic composition of West African savannas, where these families commonly structure woody communities (Ahouandjinou et al. 2017; Dongock et al. 2017; Arbonnier 2019). Furthermore, Nganso et al. (2025) reported that the Fabaceae represent the main melliferous plant family in Africa.

The results support our first hypothesis that protected area maintains the highest diversity of melliferous species. Indeed, protected area and fallow land both maintain the highest melliferous species richness, whereas farmland showed reduced species richness. The high richness observed in fallow reflects the combined effects of ecological succession and farmer‐mediated selection. With a mean duration exceeding 16 years, many fallow lands may represent advanced secondary stages approaching semi‐natural conditions (Fournier et al. 2001; Augusseau et al. 2006; Jakovac et al. 2021). Regeneration processes and the deliberate retention of culturally and economically important species such as *Parkia biglobosa* , *Tamarindus indica* , and *Vitex doniana* can generate structurally and floristically heterogeneous stands. Indeed, previous studies showed that traditional agroforestry systems in West Africa often maintain substantial woody species diversity despite continued agricultural use, as farmers retain native trees and shrubs within farmland and fallow land, supporting biodiversity conservation alongside production (Boffa 1999; Cissé et al. 2018; Zoungrana, De Cannière, et al. 2023).

Protected area showed higher melliferous species evenness, suggesting a more balanced distribution of melliferous species. This result may reflect reduced selective harvesting compared with managed lands. However, localized disturbances including edge effects, grazing, and human encroachment can influence recruitment dynamics in protected savanna ecosystems (Tranquilli et al. 2014; Komba et al. 2021; Guiatin 2025). NMDS results confirmed clear compositional differentiation among land use types, indicating that land‐use intensity and management influence melliferous species communities (Nombré et al. 2009; Montoya‐Pfeiffer et al. 2021; Birkenbach et al. 2024; Boieiro et al. 2025). Indeed, several species were significantly associated with fallow land and protected area, whereas no species was uniquely associated with farmland. This suggests that farmland assemblages largely represent subsets of those found in less intensively managed systems. Species restricted to protected area, such as *Mitragyna inermis*, are consistent with habitat specialization in gallery forests and relatively undisturbed environments (Thiombiano et al. 2012; Arbonnier 2019; Spichiger 2024).

### 4.2 Woody Stand Structure of Melliferous Communities Across Land Use Types

Woody stand structure differed significantly among land use types, thereby supporting our second hypothesis. Protected area maintained the highest tree densities, followed by fallow land, whereas farmland exhibited lower densities. This gradient reflects differences in land use intensity and management. In farmland, trees are selectively retained to reduce crop competition and facilitate field operations, thereby limiting recruitment and stem density (Balima et al. 2020; Zoungrana, De Cannière, et al. 2023; Guiatin and Lompo 2025). In fallow land, the cessation of cultivation allows gradual vegetation recovery, increasing stem density through natural regeneration (Fournier et al. 2001; Ky‐Dembele et al. 2019). In protected area, lower anthropogenic disturbance is associated with higher woody recruitment and the maintenance of multiple age cohorts (Nacoulma et al. 2011; Zoungrana, De Cannière, et al. 2023). However, trees in farmland exhibited greater height and stem diameter compared to those in other land use types. This result reflects the selective retention of large, economically valuable individuals, particularly *Vitellaria paradoxa* , coupled with reduced intraspecific competition under low‐density conditions (Kaboré et al. 2012; Cissé et al. 2018; Zoungrana, De Cannière, et al. 2023). Indeed, low tree density in farmland minimizes competitive interactions for soil resources, thereby enhancing individual tree growth rates (Garrity and Bayala 2019). These processes result in structurally open systems dominated by mature trees with favorable growth conditions.

Basal area did not differ significantly among land use types, suggesting that land use change redistributes woody biomass across size classes rather than reducing total stand basal area. Protected area concentrates biomass in numerous medium‐sized stems, whereas farmland allocates biomass to fewer but larger individuals. This size‐class redistribution of woody biomass has been documented across various forest and agroforestry systems, where farmer‐management practices influence stand structure without necessarily altering total biomass stocks (Djossa et al. 2008; Gotore et al. 2023). This structural contrast has important implications for floral resource distribution and accessibility. The spatial arrangement and size distribution of woody plants influence the temporal and spatial structure of floral resources available to pollinators and other fauna (Eckerter et al. 2022). Indeed, in farmland, the dispersed arrangement of large‐crowned mature trees creates a heterogeneous floral landscape that differs from the more continuous but smaller‐statured woody vegetation in protected area. Such differences in canopy structure and spatial configuration may affect pollinator foraging behavior, floral resource availability throughout the season, and the reproductive success of both cultivated and wild plant species (Sponsler et al. 2023).

### 4.3 Temporal Dynamics of Floral Resources and Implications for Honeybee Foraging

The third hypothesis, which predicted that temporal availability of floral resources would vary among land use types, was confirmed. Indeed, protected area provided longer flowering periods and higher density. *Mitragyna inermis* flowered for up to 5 months while maintaining relatively high local density. Species with extended flowering periods can help maintain forage during seasonal shortages.

Honeybee colony performance depends on both the number of floral resources and their timing. Landscapes with sequential flowering may therefore support more stable foraging conditions (Steffan‐Dewenter and Kuhn 2003). Although this study measured flowering periods and density rather than colony productivity, the prolonged flowering and high density of key species in protected areas suggest that these habitats provide relatively consistent forage within the landscape. Visitation intensity increased with floral density and was concentrated on abundant species, consistent with established ecological theory (Osborne et al. 1999; Ebeling et al. 2008). However, the melliferous importance of a species depends on local conditions, including competing floral resources, microclimate, and colony strength (Steffan‐Dewenter and Kuhn 2003; Nombré et al. 2009). The relative importance of individual species therefore varies across space and time.

The results indicate complementarity among land use types in supporting honeybee forage. Fallow land contributes higher species richness and includes economically important taxa retained by local communities. Protected area maintains dense stands and longer flowering periods. Farmland, although less dense, retains large individuals of key species capable of producing substantial floral resources. Management should therefore combine protection of melliferous habitats within protected area, particularly gallery forests, with retention and enrichment planting of priority species in agroforestry parkland to improve forage continuity and support pollination services and beekeeping in sudanian landscapes undergoing rapid land‐use change.

Future research should address remaining limitations of this study to refine understanding of land‐use effects on melliferous communities. In particular, incorporating both woody and herbaceous melliferous species would provide a more complete assessment of seasonal forage availability, while extended and repeated monitoring of bee visitation and colony performance would better capture temporal variability. Expanding analyses to multiple West African landscapes and including landscape‐scale environmental covariates would strengthen generalizability and improve inference on the ecological mechanisms linking land use intensity to melliferous community structure and function.

## Author Contributions

**Issaka Wendpanga Kanazoe:** conceptualization (lead), data curation (equal), formal analysis (equal), investigation (equal), methodology (equal), resources (equal), software (equal), supervision (equal), validation (equal), visualization (equal), writing – original draft (equal), writing – review and editing (equal). **Issa Nombré:** conceptualization (equal), funding acquisition (lead), methodology (equal), project administration (equal), resources (equal), supervision (equal), validation (equal), writing – review and editing (equal). **Adama Zoungrana:** conceptualization (equal), data curation (equal), formal analysis (equal), investigation (equal), methodology (equal), resources (equal), software (equal), validation (equal), visualization (equal), writing – original draft (equal), writing – review and editing (equal). **Joseph Issaka Boussim:** conceptualization (equal), funding acquisition (equal), methodology (equal), project administration (equal), resources (equal), supervision (lead), validation (equal), writing – review and editing (equal).

## Funding

This work was supported by Académie de recherche et d'enseignement supérieur.

## Conflicts of Interest

The authors declare no conflicts of interest.

## Data Availability

The data that support the findings of this study are openly available in Dryad at http://doi.org/10.5061/dryad.tb2rbp0hm.
